# Genetic *versus* Non-Genetic Regulation of miR-103, miR-143 and miR-483-3p Expression in Adipose Tissue and Their Metabolic Implications—A Twin Study

**DOI:** 10.3390/genes5030508

**Published:** 2014-07-09

**Authors:** Jette Bork-Jensen, Anne Cathrine Baun Thuesen, Claus Heiner Bang-Bertelsen, Louise Groth Grunnet, Flemming Pociot, Henning Beck-Nielsen, Susan E. Ozanne, Pernille Poulsen, Allan Vaag

**Affiliations:** 1 Department of Endocrinology, Diabetes and Metabolism, Rigshospitalet, DK-2200 Copenhagen N, Denmark; E-Mails: anne.cathrine.baun.thuesen@regionh.dk (A.C.B.T.); louise.groth.grunnet@regionh.dk (L.G.G.); 2 Steno Diabetes Center, DK-2820 Gentofte, Denmark; 3 Department of Clinical and Experimental Research, Glostrup Research Institute, Glostrup University Hospital, DK-2600 Glostrup, Denmark; E-Mail: clbb@novonordisk.com; 4 Center for Non-Coding RNA in Technology and Health, Science, University of Copenhagen, DK-2000 Frederiksberg, Denmark; E-Mail: flemming.pociot.01@regionh.dk; 5 Department of Pediatrics, Herlev University Hospital, DK-2730 Herlev, Denmark; 6 Diabetes Research Center, Odense University Hospital, DK-5000 Odense C, Denmark; E-Mail: henning.beck-nielsen@ouh.regionsyddanmark.dk; 7 University of Cambridge Metabolic Research Laboratories and MRC Metabolic Diseases Unit, Wellcome Trust-MRC Institute of Metabolic Science, Level 4, Box 289, Addenbrookes Treatment Centre, Addenbrooke’s Hospital, Cambridge CB2 0QQ, UK; E-Mail: seo10@mole.bio.cam.ac.uk; 8 Novo Nordisk, DK-2860 Bagsværd, Denmark; E-Mail: pepn@novonordisk.com

**Keywords:** micrornas, subcutaneous adipose tissue, human metabolism, twins

## Abstract

Murine models suggest that the microRNAs miR-103 and miR-143 may play central roles in the regulation of subcutaneous adipose tissue (SAT) and development of type 2 diabetes (T2D). The microRNA miR-483-3p may reduce adipose tissue expandability and cause ectopic lipid accumulation, insulin resistance and T2D. We aimed to explore the genetic and non-genetic factors that regulate these microRNAs in human SAT, and to investigate their impact on metabolism in humans. Levels of miR-103, miR-143 and miR-483-3p were measured in SAT biopsies from 244 elderly monozygotic and dizygotic twins using real-time PCR. Heritability estimates were calculated and multiple regression analyses were performed to study associations between these microRNAs and measures of metabolism, as well as between these microRNAs and possible regulating factors. We found that increased BMI was associated with increased miR-103 expression levels. In addition, the miR-103 levels were positively associated with 2 h plasma glucose levels and hemoglobin A1c independently of BMI. Heritability estimates for all three microRNAs were low. In conclusion, the expression levels of miR-103, miR-143 and miR-483-3p in adipose tissue are primarily influenced by non-genetic factors, and miR-103 may be involved in the development of adiposity and control of glucose metabolism in humans.

## 1. Introduction

MicroRNAs (miRNAs) target up to 60% of mammalian mRNAs [1,2] and have been demonstrated to play a key role in adipocyte differentiation, obesity, insulin sensitivity and risk of developing type 2 diabetes (T2D) in murine models [3,4,5,6]. Duan *et al.* have found single nucleotide polymorphisms (SNPs) in ~20% of human genomic pre-miRNA sequences [7]. Although it remains to be determined whether these SNPs have a functional role, some SNPs have been found to have an effect on the expression of the mature miRNA [8]. However, the relative influence of genetic and non-genetic factors on expression of most miRNAs is still not known.

Using murine models of T2D, Trajkovski *et al.* demonstrated that miR-103 silencing improved glucose homeostasis, insulin sensitivity and decreased the amount of adipose tissue [4], while Takanabe *et al.* have found miR-143 levels associated with markers of adipocyte differentiation, and up-regulated in adipose tissue of high-fat diet-induced obese mice [5]. We have previously demonstrated involvement of miR-483-3p in adipocyte development and storage capability in vitro. We also found elevated expression levels of miR-483-3p in adipose tissue from young men born with low birth weight (LBW) [9] who have a pre-diabetic phenotype [10,11]. However, the importance of miR-103, miR-143 and miR-483-3p in human glucose and lipid metabolism, and the relative impact of genetic and environmental factors in the regulation of their expression, are undisclosed.

In this study, we took advantage of our phenotypically well-characterized cohort of 244 twins to study the associations between the levels of miR-103, miR-143 and miR-483-3p in subcutaneous adipose tissue (SAT) biopsies. We studied their potential association with measures of *in vivo* glucose and lipid metabolism, as well as their potential associations to age, sex, birth weight and BMI. Importantly, using twins gives us a unique opportunity to explore the relative influence of genetic versus environmental factors on the levels of miR-483-3p, miR-103 and miR-143 in human SAT.

## 2. Experimental

Initially, a total of 606 twins were recruited in 1994 and 1995 from the population-based Danish Twin Register and examined as previously described [12]. Among these, 298 twins were reexamined in 2004–2005 with excision of SAT biopsies [13,14]. In the present study, 244 monozygotic (MZ) and same-sex dizygotic (DZ) Danish twins aged 62–83 years with adequate amounts of biopsy materials were included. Their clinical characteristics are presented in Table 1.

The study was approved by the regional ethical committee and conducted in accordance with the Helsinki Declaration.

**Table 1:** Subject characteristics.

	All	MZ	DZ	P
*n* (men/women)	244 (110/134)	96 (51/44)	148 (59/90)	-
*n* (in pairs/single twins)	156/88	66/30	90/58	-
Age (years)	73.4 ± 5.2	73.8 ± 4.9	73.2 ± 5.4	0.36
BMI (kg/m^2^)	26.0 ± 3.7	26.0 ± 3.2	26.1 ± 4.0	0.97
Birth weight (g)	2641.0 ± 446.7	2608.6 ± 469.7	2659.8 ± 431.9	0.50
2 h OGTT glucose (mmol/L)	8.3 ± 3.8	8.0 ± 3.5	8.5 ± 4.0	0.37
Triglycerides (mmol/L)	1.3 ± 0.6	1.4 ± 0.7	1.2 ± 0.5	**0.02**
HOMA-IR	1.8 ± 1.3	1.9 ± 1.7	1.8 ± 1.1	0.53
HbA1c (%)	5.8 ± 0.7	5.8 ± 0.6	5.8 ± 0.7	0.89
miR-103 (RQ)	2.1 ± 1.8	1.8 ± 1.7	2.3 ± 1.9	**0.03**
miR-143 (RQ)	3.2 ± 2.1	3.4 ± 2.3	3.2 ± 2.0	0.54
miR-483-3p (RQ)	2.5 ± 1.9	2.5 ± 1.8	2.5 ± 1.9	0.88
*n* (diabetes)	31 (13%)	9 (9%)	22 (15%)	-
*n* (IGT)	72 (30%)	32 (33%)	41 (28%)	-
*n* (NGT)	139 (57%)	54 (56%)	85 (57%)	-

Values are shown as mean ± standard deviation. Differences between monozygotic (MZ) and dizygotic (DZ) twins were tested using Welch’s two sample *t*-test in R. *p* ≤ 0.05 was considered significant and displayed in bold. Body mass index (BMI), plasma glucose measured at 2 h in an oral glucose tolerance test (2 h OGTT glucose), homeostatic model assessment of insulin resistance (HOMA-IR), hemoglobin A1c (HbA1c), relative normalized quantities of miRNA (RQ). Normal glucose tolerance (NGT) was defined as 2 h glucose <7.8 mmol/L, impaired glucose tolerance (IGT) was defined as 2 h glucose ≥7.8 mmol/L and <11.1 mmol/L. Diabetes was defined as 2 h glucose ≥11.1 mmol/L.

### 2.1. Clinical Examination and Tissue Sampling

All subjects underwent a 75 g standardized oral glucose tolerance test (OGTT). Plasma glucose, insulin and triglyceride concentrations were analyzed as previously described [12]. Of the 244 subjects 13% had diabetes, 30% had impaired glucose tolerance and 57% had normal glucose tolerance. Insulin resistance was determined using homeostatic model assessment of insulin resistance (HOMA-IR) [15]. SAT biopsies were collected from the abdomen as previously described [13].

### 2.2. miRNA Expression Analysis

Total RNA was extracted using miRNeasy kit (Qiagen, Hilden, Germany). cDNA was synthesized using Taqman^®^ MicroRNA Reverse Transcription Kit and pooled stem-looped primers for RNU48, hsa-miR-483-3p, hsa-miR-103 and hsa-miR-143 (Applied Biosystems (ABI), Foster City, CA, USA). Triplicate cDNA samples were quantified with the corresponding Taqman^®^ MicroRNA Assays (ABI) using the BioRad CFX384 real-time PCR system (BioRad Laboratories, Hercules, CA, USA). miRNA expression levels were normalized to RNU48, which was unaffected by any of the investigated parameters.

### 2.3. Statistical Methods

Comparisons between MZ and DZ twins (Table 1) were performed using Welch’s two-sample *t*-test in the statistical program R. Multivariate analyses were performed using the PROC MIXED procedure in SAS v9. All analyses performed on the total cohort were adjusted for age, sex, BMI, zygosity and twin pair. Analyses performed to examine the effect of age, sex and BMI were additionally adjusted for birth weight. Analyses involving metabolic parameters were conducted on the total cohort and separately for MZ and DZ twins. The analyses stratified for zygosity were adjusted for BMI, age, sex, and twin pair. Response variables were log_e_-transformed. The calculated effects were transformed into percent-wise changes per standard deviation (SD). Results are shown with 95% confidence intervals. *p* ≤ 0.05 was considered significant.

The following analyses were all performed in R. Interclass correlations for MZ (r_MZ_) and DZ (r_DZ_) twins were calculated with 2n as previously recommended [16] using Spearman’s rank correlation coefficient. The interclass correlation coefficients were compared using Fisher z-transformation and heritability was calculated as [h^2^ = 2(r_MZ_ − r_DZ_)]. *p*-values were calculated with 1n to avoid exaggerated power.

## 3. Results and Discussion

### 3.1. Age, Sex and Anthropometric Measurements Associated with miRNA Expression in Adipose Tissue

**Figure 1:**
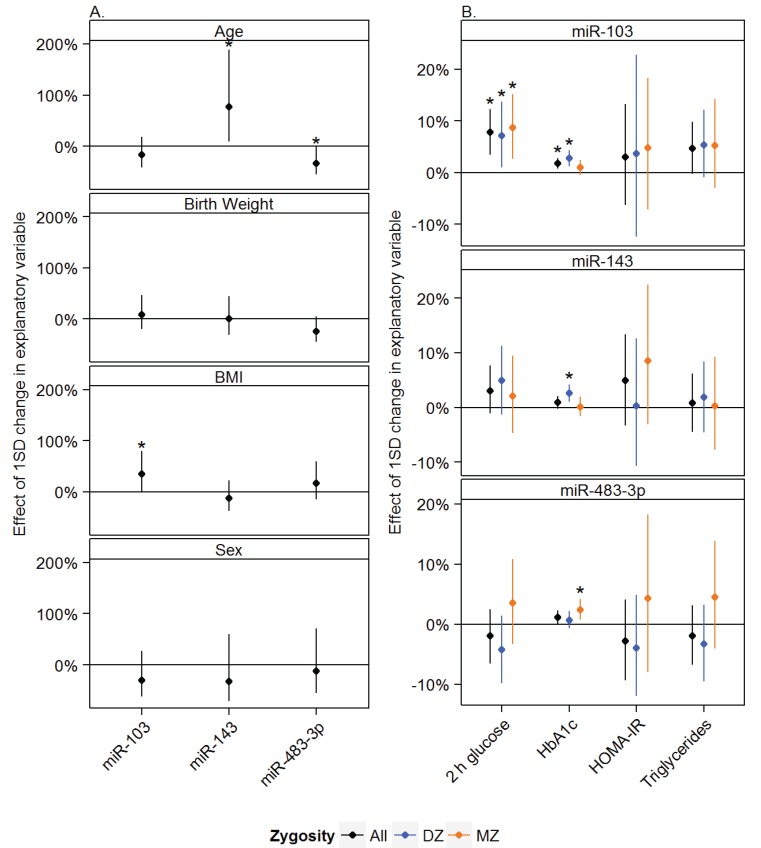
Multivariate analyses were performed to study the association between (**A**) age, birth weight, BMI and sex and miR-103, miR-143 and miR-483-3p expression in adipose tissue from elderly twins and the association between (**B**) miR-103, miR-143 and miR-483-3p and 2 h glucose values after an oral glucose tolerance test, hemoglobin A1c (HbA1c), homeostatic model assessment of insulin resistance (HOMA-IR) and triglycerides shown for all subjects (black), dizygotic (DZ) twins (blue) and monozygotic (MZ) twins (orange). Effect measures were transformed to percent wise changes per standard deviation (SD). Mean effects are shown by ●. Error bars represent the 95% confidence interval. All analyses were adjusted for age, sex and BMI. In addition the analyses of age, BMI and sex were adjusted for birth weight. Analyses involving all subjects (black) were additionally adjusted for twin and zygosity status. A *p*-value of ≤0.05 was considered significant, and is indicated by *. Body mass index (BMI), plasma glucose measured at 2 h in an oral glucose tolerance test (2 h glucose), homeostatic model assessment of insulin resistance (HOMA-IR), hemoglobin A1c (HbA1c).

We evaluated the associations between age, sex, birth weight and BMI; and the expression levels of miR-483-3p, miR-103 and miR-143 (Figure 1A). *Age*: We found that a ~5 year increase in age was associated with a ~33% decrease in miR-483-3p levels (*p* = 0.05) and with a 77% increase in miR-143 levels (*p* = 0.02). *Sex*: There were no statistically significant associations between sex and the expression levels of the miRNAs. *Birth weight*: We found a trend towards an association between miR-483-3p levels; a ~450 g increase in birth weight was associated with a 24% decrease in miR-483-3p levels. This effect was, however, not statistically significant (*p* = 0.09). *BMI*: We found that an increase of 3.7 kg/m^2^ in BMI was associated with a 34% increase in miR-103 expression (*p* = 0.05).

### 3.2. Associations between miRNAs and Measures of Glucose and Lipid Metabolism

We evaluated the associations between glucose metabolism (2-hour (2 h) glucose from an OGTT and hemoglobin A1c (HbA1c)), insulin resistance (HOMA-IR) and lipid metabolism (triglyceride levels); and miR-483-3p, miR-103 and miR-143 expression levels (Figure 1B). *miR-483-3p:* An increase of one SD (~10%) in miR-483-3p levels was borderline significantly associated with a 1% increase in HbA1c (*p* = 0.06). miR-483-3p was not statistically significantly associated with 2 h glucose levels, triglycerides or HOMA-IR in the total cohort. In the MZ twins, increased miR-483-3p levels were associated with increased HbA1c values. *miR-103*: A one SD (~10%) increase in miR-103 levels was associated with a ~8% increase in 2 h glucose levels (*p* < 0.001), a 2% increase in HbA1c (*p* = 0.001) and, additionally, we found a trend towards a 5% increase in triglycerides (*p* = 0.07). No statistically significant association was found to HOMA-IR. *miR-143*: We did not find any statistically significant associations between miR-143 and any of the investigated metabolic parameters in the total cohort. In the DZ twins we found a statistically significant positive association between the levels of miR-143 and Hba1c levels.

### 3.3. Effects of Zygosity

Triglycerides were significantly higher in the MZ twins, a difference that has previously been shown in this cohort [17]. Dizygotic twins had higher levels of miR-103 compared to the MZ twins (*p* = 0.03) (Table 1). The effect of zygosity was still present when adjusted for age, sex and BMI in the multivariate analysis but only with a borderline statistical significance (*p* = 0.06). There were no differences or associations between zygosity and miR-143 or mir-483-3p levels. In general, there were few differences between MZ and DZ twins in the separated multivariate analyses (Figure 1B).

### 3.4. Heritability Analysis

The interclass correlations for MZ and DZ twins were not significantly different for any of the miRNAs and all heritability estimates were low (0.21, 0.12 and ~0 for miR-483-3p, miR-143 and miR-103 respectively).

### 3.5. Discussion

In this study we aimed to investigate factors which may regulate the expression of three miRNAs: miR-483-3p, miR-103 and miR-143, as well as the association between these miRNAs and measures of human glucose and lipid metabolism. Previous studies have shown that SNPs in pre-miRNA transcripts are involved in the regulation of mature miRNA levels [18]. Using SAT biopsies from a unique cohort of 244 elderly MZ and DZ twins, we estimated the genetic influence on the expression of miR-483-3p, miR-103 and miR-143, and found low heritability estimates. This indicates that environmental factors are the major determinants of the expression of the miRNAs investigated in this study.

We previously reported that LBW is associated with increased levels of miR-483-3p, which may lead to decreased adipocyte expandability through down-regulation of growth differentiation factor-3. This may contribute to insulin resistance through decreased lipid storage capacity resulting in increased circulating lipids and ectopic lipid storage [9]. Thus, miR-483-3p could be an important link between poor intrauterine environment and later development of T2D. In our current study of elderly twins, we found the same trend in the association between miR-483-3p and birth weight although it did not reach statistical significance. In the MZ twins we found a positive association to HbA1c, however this association was only borderline significant in the total cohort. This may indicate a role in glucose metabolism, but as we did not find the expected association between miR-483-3p expression levels and HOMA-IR as a measure of insulin resistance, further studies will be needed to understand the specific role of this miRNA in human metabolism. The level of *IGF2*, the host gene of miR-483-3p, is known to decline with age [19] and in accordance with this we observed decreased expression of miR-483-3p with increasing age. We suggest that the age-associated silencing of this locus masks the effects of the intrauterine programming over time, possibly in conjunction with the altered adipose tissue function and distribution with increasing age. Furthermore, the high age of the twin cohort may have introduced a selection for individuals with the least adverse metabolic manifestations, and moreover, the lower birth weight of twins compared to singletons [20] may influence the association.

Trajkovski *et al*. demonstrated that miR-103 silencing improved glucose homeostasis and insulin sensitivity in mice and demonstrated an association between miR-103 and body weight [4]. miR-103 has also been investigated in two relatively small human studies, with no detected differences in the expression between non-obese and obese women with or without T2D (*n* = 6–13) [21], or between newly diagnosed T2D subjects and control subjects (*n* = 6–9) [22]. Considering the variation in human subjects, we expected that possible effects upon miR-103 expression might more readily be revealed through investigation of a larger cohort. The results by Trajkovski *et al*. suggest that miR-103 repression of caveolin-1, through destabilization of the insulin receptor, leads to increased insulin resistance in the adipose tissue [4]. This could lead to increased lipolysis, elevated circulating free fatty acid levels and progression of whole body insulin resistance. In the present study of 244 subjects, we found that miR-103 levels were positively and independently associated with BMI, 2 h glucose levels as well as HbA1c, and, in addition, we found a trend towards a positive association with plasma triglyceride levels. Therefore, although our current cross-sectional association study cannot establish causality, we suggest that miR-103 is likely to be involved in the development of a dysfunctional metabolism and possibly T2D in humans. While we did not see any association between miR-103 and insulin resistance as expressed by HOMA-IR, it should be noted that a more specific measure of insulin resistance, which can be obtained using the euglycemic hyperinsulinaemic clamp technique, was not available.

Jordan *et al.* demonstrated that increased expression of miR-143 in the liver could induce insulin resistance through down-regulation of ORP8, subsequently preventing AKT activation by insulin [6]. As ORP8 is also present in adipose tissue [23], the same mechanism may also exist here. However, our study revealed no associations between miR-143 and HOMA-IR. Except for a significant positive association between miR-143 levels and Hba1c, we did not find any other measures that would support an involvement of adipose tissue miR-143 in human metabolism.

We found that miR-103 was present in higher levels in the DZ twins compared to the MZ twins. As zygosity is known to influence metabolism [24] we stratified the association analyses between the miRNAs and metabolic measures by zygosity. This allows for analysis of trends in the separate groups, but also reduces the power of the analysis, thereby increasing the risk random findings. Therefore, these results should be interpreted with caution.

## 4. Conclusions

In conclusion, we have demonstrated that the expression levels of miR-483-3p, miR-103 and miR-143 in SAT are mainly influenced by age, obesity and birth weight in a non-genetic manner. miR-103 has previously been found to have a key role in murine insulin sensitivity, and our findings here suggest that miR-103 may have a similar importance in human adiposity and glucose metabolism.
